# The Public Health: Interviews with Local Authorities

**Published:** 1922-09

**Authors:** 


### THE PUBLIC HEALTH.

### INTERVIEWS WITH LOCAL AUTHORITIES.

### III.—THE CITY OF BIRMINGHAM.

"THERE be many smithes in the towne that use to make knives and all maner of cuttyng tools and many lorimars that make bytes." So wrote John Leland in the year 1540 in the earliest description which exists of the place then known as "Bremischam "——" A good market towne whose beauty is in one strete goynge up almost from the left ripe of the broke up a mene hill by the length of a quarter of a mile."

If John, or his shade, were to revisit Birmingham now, he would learn that there are still to be found there many smithes and lorimars and indeed other manner of cunning workmen. Vast numbers, in fact, occupying an area extending 16 miles in one direction, and 12 in another, and covering 43,000 acres of ground. And it is the business of those in authority in the City of Birmingham to see that these vast numbers of workmen with their dependants—comprised in a population now numbering 920,000—are well housed and care for.

Fortified with the knowledge that another visitor to Birmingham in 1752, then a considerable market town, found its inhabitants to be "generally of an obliging and ingenious disposition," we sought an interview with Mr. Lucas, the Chairman of the Public Health Committee, and Dr. Beazeley, the Deputy Medical Officer of Health, and found that these kindly characteristics still obtained.

#### The Milk Supply.

Our Medical Officer, Dr. Robertson, they said, is in America, studying, amongst other things, certain aspects of the problem of a pure milk supply, and we may give you some indication of what we are already doing in this direction. There were 142 dairy farms in the city at the end of the year 1921, having 276 registered cowsheds, housing 1,890 dairy cows. It is gratifying to be able to say that there was no case of a cow affected with tuberculosis of the udder found in any of the city dairies during the year. In 20 farms, on notice being given by our officers, necessary repairs and alterations to cowsheds were carried out to enable the owners to have their names retained on the register.

Of 184 samples of milk arriving from outside sources by lorry and train, 9 were found to contain living tubercle bacilli. The farms in these cases were visited, further samples taken and, in the result, eight cows were slaughtered.

Outside the city, 16 herds comprising 483 cows supplying milk to the city, were being dealt with under our scheme. All but one herd of 19 cows were free from tubercle bacilli, and that herd was being freed. The retailers of milk are especially attended to, and in no less than 56 cases were prosecutions for adulteration considered necessary in 1921, fines amounting in all to £210 being imposed.

#### Tuberculosis.

The control of the milk supply is one only of the measures in our campaign against tuberculosis. That the preventive work on which we are engaged is bearing fruit, is shown by the marked decline in the number of new cases of tuberculosis notified in 1921. From a total number of 3,000 new notifications in 1915, the yearly number has dropped to under 2,000 in 1921; the deaths in the same period have fallen from 1,141 to 890. This is worth while coupling with the interesting fact that our notifications per 100 deaths continue to be more numerous than in any other of the large cities.

*A History of Cases treated in* 1914.—A report was issued in 1920 by the Birmingham Insurance Committee tracing the history of the insured persons who had been recommended for sanatorium benefit in 1914. The summary of findings contains the following:—

"A death rate of 50 per cent. in six years, with retention of full working capacity in 72 per cent. of the survivors, whilst much better than the results obtained in areas where the problem has been dealt with half-heartedly, leaves much margin for improvement."

It will be seen that it is claimed on our behalf that we have not been half-hearted in this matter. Our gross expenditure on tuberculosis in fact grew from £7,789 in the year 1911 to £105,000 in 1920.

*An Unexplained Fact.*—The morbidity rates taken on an average of seven years are 400 males per 1,000 cases notified, as compared with 300 females per 1,000. It is an interesting fact for which we are unable to give an adequate explanation.

*The Work of the Tuberculosis Visitor.*—This is an important part of the work of tuberculosis prevention. During 1921, 2,340 primary visits were paid to new cases, 31,755 periodical re-visits (old and new cases), and 11,921 special re-visits. The first duty of the visitor is to see that the patient is taking all reasonable precautions to prevent the spread of infection. This involves rearrangement of sleeping accommodation so as to ensure that the patient will not share a bed, nor, if possible, the same bedroom with any other member of the household. Where a patient is unable to provide a separate bed, and the financial conditions of the family warrant such assistance, bed and bedding are provided either on loan or on a system of hire-purchase with repayments spread over a long period. In 1920, 390 beds were distributed under this scheme, including 70 purchased on the instalment system. For the same year, as a direct result of this work, 541 patients who were found on a previous visit to be sharing a bed, had made arrangements to sleep alone, and 266 patients who were sharing the same bedroom with other members of the family, were found on re-visiting to be occupying separate bedrooms. In suitable cases recommendation is made for the provision of shelters to be erected in the garden. The number of shelters in use at the end of the year was 132, of which 51 were occupied by ex-Service men and 81 by other cases. The method of the disposal of sputum is also carefully supervised, and patients are advised verbally and by leaflets how best to keep well.

*Educational Work.*—We believe that this work of spreading knowledge, not only amongst tuberculous persons and their "contacts" (who present themselves for examination in increasing numbers), but amongst the whole community, is a most important aspect of public health work. One difficulty which must be frankly faced is that what is necessary to appeal to the ignorant and thriftless is considered superfluous when addressed to those already educated in more general matters. But this educational work ranks high in our estimation with such things as good drainage and good water.

*Sanatorium Treatment.*—Between 500 and 600 patients are undergoing treatment in our sanatoria at any time, and we give special attention to early cases, said Mr. Lucas, and get them away to Salterley Grange to breathe the healing air of the Cotswold Hills, and aim at giving a prolonged stay in all cases, where the patient is willing, which give promise of favourable results.

*Powers to Isolate.*—We possess, under an Act of 1914, the power to isolate compulsorily persons suffering from tuberculosis who are a danger to the community. It is a power which would have to be exercised, however, with great caution, and it is enough to say that at present we regard it as a means of bringing pressure to bear when it is difficult to get cases out of surroundings in which they are an acute danger to those about them.

### Child Welfare.

Until about 20 years ago, practically nothing was done to reduce child mortality in this country. In Birmingham the practical result of our work in the last 20 years is to reduce child mortality to about one-half, that is to say, compared with 20 years ago, *there is a saving in the normal year of* 3,000 *lives.* The work of establishing centres has been developed until every suitable district is covered. £46,500 was spent in child welfare work in the 12 months ending March 31, 1921, and those who question whether adequate results are obtained for this outlay are not sufficiently appreciative of the fact that with the care and development of the child is wrapped up the whole of the city's future, and that, as with all educational work, the results are not immediately apparent. The Carnegie Trust, at any rate, recognised the wisdom of child welfare expenditure, and made a grant for this purpose to the city of no less than £25,000.

It is not infrequently contended that the improvement in the infant mortality rates has been entirely due to the fact that there has been a series of cold summers. This is not borne out by the Birmingham statistics, which show that the infant mortality from causes other than diarrhœa and enteritis has been reduced at almost the same rate as the total mortality. This was pointed out by Dr. Robertson in 1921, and it will be noted that the rate since ascertained for that year (with a hot summer) agrees with the low rate of 1920, *i.e.*, 83 per 1,000 births, the lowest recorded in Birmingham, and lower than the rate for most of the large cities.

### Housing.

The population of Birmingham, as shown by the census figures, has increased nearly 10 per cent. in the last 10 years (as compared with a general rate of increase in the country of 5 per cent.), but so far from keeping pace with this extraordinary increase, the housing provision has fallen away badly. The number of new houses per 100,000 of the population fell away between 1901 and 1910 from 447 to 230, and practically ceased altogether in 1918 and 1919. In 1920 the number was 72, and in 1921, 153.

The work of demolition of slum properties and reconstruction of others is crippled because there are no new houses for the people to move into. An interesting experiment is, however, being carried out in a small way, by which the Municipality provides a few "accommodation" houses, into which families are temporarily moved while their own homes are being reconstructed, and then move out for others similarly to enter into temporary occupation. It is shown by a recent return that whereas there were 9,755 empty houses in 1912, many of them unfit for human dwellings, there are now none. They have been filled up, not demolished, and the overcrowding is most serious.

A great deal of this congestion has arisen in our own lifetime, said Mr. Lucas. Within a half-mile of my own factory, in the centre of the city, I used, as a boy, to gather blackberries, and amongst the rules of the General Hospital (little more than a stone's throw from the City Hall) is one which prohibits rabbit shooting in the adjacent warrens.

We feel certain that if only dwellers in the crowded central areas can be moved from their pernicious environment into decent surroundings, their habits of life will be vastly improved. As a Health Committee we cannot move except by bringing pressure on the Housing Committee.

Other health matters of interest were covered in the course of our interview, but we close this account on the note of housing. It is clear that Birmingham needs an extensive housing programme in order that her great army of workers may be properly and decently housed. The death-rate has already fallen by more than one-half in 50 years, from 25·2 in 1871 to 11·3 in 1921. And a housing programme carried out on a bold plan is not going to make much difference in the rate at which the people die. But it is going to make a lot of difference in the way in which some of the people live.

Birmingham has done big things in the past. Do we not think of her as the home of municipal enterprise ? Some of us have been accustomed to think that, in the growth of a sensitive social feeling, and of a demand that organised and responsible measures shall be substituted for isolated and spasmodic effort, the influence of the City fathers of Birmingham has been at work.

There is something inspiring in the fact that Birmingham went into a remote district of Wales with a population of less than 100 souls, exclusively sheep farmers, their families and shepherds, and built vast masonry dams and reservoirs, and gathered water there, and constructed 73 miles of aqueduct through which pour the daily supplies of pure water for her vast industrial population. Yes, and it cost money—£6,000,000, no less. Is it too much to hope that Birmingham can tackle her housing difficulties in the same inspiring way ?

